# Developmental trajectories of and reciprocal relationships between Chinese university students' foreign language reading self-efficacy and reading strategy use

**DOI:** 10.3389/fpsyg.2025.1512098

**Published:** 2025-04-11

**Authors:** Shiyu Zhou, Yingxian Zhang

**Affiliations:** ^1^School of Foreign Languages, China University of Geosciences (Wuhan), Wuhan, China; ^2^School of Foreign Languages, Huazhong University of Science and Technology, Wuhan, China

**Keywords:** foreign language reading self-efficacy, foreign language reading strategy use, developmental trajectories, predictive relationships, EFL education

## Abstract

Despite extensive research recognizing the critical role of foreign language reading self-efficacy and reading strategy use in L2 learning, longitudinal studies examining the relationships between these two variables from a dynamic developmental perspective remain scarce. This study investigated the developmental trajectories and reciprocal predictive relationships between foreign language reading self-efficacy and reading strategy use within the context of English as a foreign language (EFL) education in China. Data were collected from 293 Chinese undergraduate EFL students at five time points over the course of one academic year using a mixed-methods approach which included parallel latent growth models, cross-lagged analyses, and semi-structured interviews. Quantitative analyses indicated that the participants' foreign language reading self-efficacy firstly increased, followed by a decline, and then rose again, with significant individual variations in both initial levels and rates of change. The initial levels did not affect the rates of increase. Meanwhile, the participants' use of foreign language reading strategies showed a significant increase over time. The initial levels of reading strategy use did not influence its rates of change, with only the former exhibiting significant individual differences. Moreover, positive correlations were found between the initial levels of reading self-efficacy and reading strategy use, as well as between their growth patterns. Additionally, a bidirectional predictive relationship was identified between foreign language reading self-efficacy and reading strategy use, with the influence of reading strategy use on self-efficacy being stronger than the reverse. Qualitative results provided further insights into the participants' changes in their reading self-efficacy and the underlying factors driving these changes. The findings hold practical implications for EFL educators, highlighting the necessity of incorporating self-efficacy-enhancing instruction and reading strategy training in their reading classes.

## 1 Introduction

Reading is an essential skill for English as a Foreign Language (EFL) learners, and foreign language reading comprehension is a dynamic and multifaceted process shaped by “linguistic and cognitive, social and cultural, affective and motivational factors” (Li and Wang, 2010, p. 145). Impacted by positive psychology, the importance of positively affective and cognitive experience has gained increasing recognition in the field of foreign language learning (e.g., Dewaele and Li, 2020; Elahi Shirvan et al., 2020, 2021).

As an important variable in L2 learning, self-efficacy refers to an individual's belief and confidence in their capacity to successfully perform a task or attain a specific goal (Bandura, 2001). It can have beneficial effects on foreign language learning, such as reducing foreign language anxiety, enhancing proficiency, improving learning outcomes, and encouraging greater efforts, all of which contribute to better performance in English reading comprehension (Elahi Shirvan et al., 2018; Liu and Dong, 2023; Oh, 2016). Previous studies have explored the relationship between reading self-efficacy and reading performance. For instance, Li and Gan (2022) demonstrated that reading efficacy positively predicted Chinese EFL students' English reading comprehension, supporting the notion that students with stronger beliefs in their reading abilities tend to achieve better reading outcomes. Yang et al. (2024) reported that most Chinese university students exhibited moderate to medium-low levels of reading self-efficacy. Their findings indicated that while high self-efficacy students showed a positive correlation between self-efficacy and reading performance, no significant relationship was observed for students with low self-efficacy.

Foreign language reading is also shaped by the reading strategies learners employ, with frequent use of the strategies serving as a strong predictor of foreign language reading performance (Shang, 2010). Given the significance of foreign language reading self-efficacy and reading strategies, scholars have turned their attention to exploring the connections between these two variables. Recent research has provided evidence that reading self-efficacy significantly influenced the use of various reading strategies. Yao (2023) identified a strong and statistically significant correlation between reading strategies, motivation, and self-efficacy among Chinese EFL college students. Li et al. (2024) found that reading efficacy predicted the use of control strategies, memorization strategies, and elaboration strategies among Chinese junior secondary students, while also predicting control strategies among Chinese senior secondary students. These findings suggest that students with higher reading self-efficacy are more likely to employ a diverse range of reading strategies, reinforcing the well-established link between self-efficacy and strategic learning behaviors. Alsuhaibani (2019) used questionnaires and retrospective semi-structured interviews to demonstrate that reading self-efficacy could significantly and positively predict the use of EFL reading strategies. Shehzad et al. (2020) study on 188 Saudi EFL university learners reached a similar conclusion. While prior studies have extensively examined how reading self-efficacy influences reading strategies, less attention has been given to the possibility of a reciprocal relationship—whether learners' use of foreign language reading strategies can, in turn, predict their foreign language reading self-efficacy. Understanding this bidirectional relationship is crucial, as it can provide deeper insights into how reading strategy instruction may enhance students' confidence in reading. Therefore, further investigation into the predictive effects of reading strategies on reading self-efficacy is warranted.

Moreover, Complex Dynamic Systems Theory (Larsen-Freeman and Cameron, 2008) suggests that the systematicity and interactivity of variables should be examined from a dynamic developmental perspective, which provides important insights into understanding the emotional changes and cognitive development of foreign language learners. In addition, China has the largest population of EFL learners, whose diverse cultural backgrounds, motivations, self-efficacy, and learning strategies (Tao and Yu, 2024) reflect challenges that are not unique to Chinese learners but are shared across global EFL contexts. Research has shown that issues such as building confidence in reading tasks (Tangkijmongkol and Wasanasomsithi, 2022), employing effective reading strategies (Muche et al., 2024), and overcoming language anxiety (Çapan and Pektaş, 2013) are common among EFL learners worldwide. The interaction between reading self-efficacy and strategy use, as observed in Chinese EFL learners, provides a valuable lens through which to understand these global challenges. By exploring this dynamic relationship in the context of one of the largest EFL learner populations, this study can contribute to a broader understanding of how foreign language reading self-efficacy and strategy use develop and interact in diverse educational settings. Therefore, this study aims to explore the dynamic developmental trajectories and reciprocal predictive relationships between Chinese university students' foreign language reading self-efficacy and use of foreign language reading strategies through a longitudinal survey, with the hope of providing empirical evidence and pedagogical suggestions for emotional intervention and cognitive development in foreign language teaching and research.

## 2 Literature review

### 2.1 Foreign language reading self-efficacy

Reading self-efficacy generally refers to students' belief in their ability to effectively accomplish different reading tasks (Yang and Gan, 2024). In this study, foreign language reading self-efficacy was defined as learners' perceptions of their capability to perform various reading tasks in L2 classes (Li and Wang, 2010). In the study of 182 sophomore English majors in a university in China, Li and Wang (2010) concluded that the students generally felt rather confident of their abilities to perform English reading tasks measured by the English reading self-efficacy questionnaire. Elahi Shirvan et al. (2018), in their longitudinal study involving 367 Iranian undergraduate students over four time points within a semester, reported a significant increase in students' self-efficacy.

However, not all researchers found a steady increase in students' reading self-efficacy. For instance, Wijaya (2021) noted that Indonesian English Education master students experienced a decline in their perceptions of self-efficacy in academic reading over one semester. Li et al. (2022) investigated the effects of reading strategy instruction on Chinese university EFL students' reading self-efficacy. Their findings revealed that, despite improvements in reading comprehension, the students' reading self-efficacy showed no significant changes. The researchers suggested that this might be attributed to the relatively short duration of the intervention, which may not be sufficient to bring about substantial changes in self-efficacy. They further proposed that noticeable motivational or attitudinal transfer effects of reading strategy interventions were more likely to occur in programs lasting more than 1 year.

The conflicting findings indicate the intricate dynamics of foreign language learners' reading self-efficacy. Moreover, since all of the above-mentioned studies are cross-sectional, longitudinal studies are needed to trace learners' reading self-efficacy over a prolonged period.

### 2.2 Foreign language reading strategies

Reading strategies were defined as “deliberate, conscious procedures used by readers to enhance text comprehension” (Li and Wang, 2010, p. 146). Research examining the impact of reading strategy instruction on L2 reading comprehension and strategy use has generally demonstrated positive effects. For instance, Shih et al. (2018) found that combining reading strategy instruction with extensive reading could improve 10th-grade students' English reading comprehension and enhance their application of reading strategies. During their 15-week reading strategy intervention, Liao and Wang (2018) found that the experimental group receiving the intervention outperformed the control group in both reading comprehension and the frequency of reading strategy use. In a one-year experimental study, Yapp et al. (2021) observed a significant increase in the use of L2 reading strategies among first-year Dutch polytechnic students (*p* < 0.001). Several studies have also indicated that reading strategy instruction did not have a significant effect on students' reading strategy use. Shang (2010) explored the frequency of undergraduates' reading strategy use during a whole-semester instruction of reading skills. The analysis of the reading strategy questionnaires administered at the start and the end of the semester illustrated that the students generally used reading strategies more frequently, although no significant differences were identified at the 0.05 probability level. Zenotz (2012) implemented an online reading strategy instruction in Spain to examine potential changes in the use of reading strategies and reading comprehension among EFL university students. The findings indicated that while the metacognitive strategy training positively influenced online reading, it did not impact the quantity of strategies employed. The conflicting findings of the previous studies suggest a need to further investigate the developmental trajectory of foreign language reading strategy use.

### 2.3 Foreign language reading self-efficacy and reading strategy use

The results of existing research indicate positive relationships between the reading self-efficacy of foreign language learners and the frequency of reading strategies use by collecting cross-sectional data. Researchers have examined the link between foreign language learners' reading self-efficacy and their use of different reading strategies, including metacognitive strategies and cognitive strategies (e.g., Alsuhaibani, 2019; Shehzad et al., 2020), compensation strategies (e.g., Ghezlou and Biria, 2012), and word-process strategies (e.g., Leung et al., 2019). In their cross-sectional survey, Li and Wang (2010) found that reading self-efficacy among sophomore English majors in one semester was significantly positively correlated with their overall reading strategy use (*r* = 0.36, *p* < 0.01). Readers with high self-efficacy reported using reading strategies significantly more often than those with low self-efficacy. In addition, studies employing regression analysis observed the predictive effect of reading self-efficacy on reading strategy use (e.g., Alsuhaibani, 2019; Leung et al., 2019; Shehzad et al., 2020). Alsuhaibani (2019) used questionnaires and retrospective semi-structured interviews to demonstrate that reading self-efficacy could significantly and positively predict the use of EFL reading strategies. Shehzad et al. (2020) conducted a study with 188 Saudi EFL university learners and arrived at a similar conclusion. Leung et al. (2019) monitored the eye movements of Japanese EFL learners and found that students with greater reading self-efficacy tended to employ global strategies more often in reading tasks, whereas those with lower reading self-efficacy were more inclined to use local strategies.

The question of whether learners' use of foreign language reading strategies can also predict their foreign language reading self-efficacy remains relatively underexplored. One of the few studies to address this issue is Liao and Wang's (2018) research, which found that the use of comprehension strategies significantly predicted an increase in students' English reading self-efficacy. Similarly, Graham et al. (2020) investigated beginner learners of French and found that text engagement regulatory reading strategies (TERRS) served as an important predictor of reading self-efficacy. In another study, Yao (2023) used a descriptive correlational approach and concluded that Chinese non-English major college students who demonstrated improved reading tactics also reported enhanced reading motivation and self-belief.

Nevertheless, the existing literature has not yet examined how durable self-efficacy gains might be over time. This gap highlights the need to explore whether the relationship between reading strategies and self-efficacy is bidirectional, rather than unidirectional. Specifically, it remains unclear whether improvements in reading strategy use can lead to long-term enhancements in reading self-efficacy and vice versa. Furthermore, the majority of previous studies have been cross-sectional, limiting our understanding of how these two constructs interact over extended periods. Therefore, longitudinal research is essential to uncover the dynamic and evolving nature of the relationship between foreign language reading self-efficacy and reading strategy use.

### 2.4 A complex dynamic system theory (CDST) perspective on foreign language reading self-efficacy and reading strategies

Complex Dynamic Systems Theory (Larsen-Freeman and Cameron, 2008) provides a new research perspective on the interaction between the emotional and cognitive development of foreign language learners. This theory posits that most individual factors are not static but change over time, with different factors existing within different subsystems and constantly interacting with each other (Elahi Shirvan et al., 2021). This applies to learners' foreign language emotions as well as their ability to use reading strategies. However, scant research has explored the relationship between foreign language reading self-efficacy and strategy use, especially from a CDST perspective. This gap in prior research forms the basis of the current study.

Some of the few studies addressing foreign language reading self-efficacy (Kyo, 2022; Elahi Shirvan et al., 2018; Yang et al., 2024) and reading strategies (Xu et al., 2017; Chen, 2019; Dong and Liu, 2022) provide valuable insights. Based on the theoretical framework of CDST, Kyo (2022) employed Latent Growth Curve Modeling (LGCM) to investigate the developmental features of reading self-efficacy among secondary school students in South Korea over 3 years and concluded that the students' L2 reading self-efficacy demonstrated a slight increase over time. The findings revealed that L2 reading self-efficacy was not a stable trait but an evolving construct, reflecting CDST's emphasis on change and adaptability over time. By administering questionnaires at four time points with two-week intervals during one semester, Elahi Shirvan et al. (2018) similarly applied LGCM to analyze the self-efficacy of undergraduate students in Iran. The results demonstrated significant heterogeneity in students' growth trajectories, illustrating CDST's principle of variability and the dynamic interaction of individual and contextual factors. Yang et al. (2024) identified four distinct profiles of reading self-efficacy among Chinese EFL learners using latent profile analysis, highlighting the influence of cultural norms, such as modesty, on students' self-efficacy.

Most CDST studies have focused on writing (e.g., Speelman and Verspoor, 2010; Verspoor et al., 2008) and listening strategies (Dong, 2016), while foreign language reading strategies remain underexplored. Xu et al. (2017) conducted a review of studies on foreign language reading strategies, emphasizing the need to understand reading strategies as a complex and dynamic system and highlighting the paucity of research on their longitudinal development among EFL learners. Chen (2019) addressed this gap by employing the moving min-max graph and Change-Point Analyzer to examine the developmental trajectories of Chinese undergraduates' reading strategy use. The findings revealed a non-linear pattern with a sharp increase during the midterm examination, reflecting CDST's focus on phase shifts triggered by external conditions. Dong and Liu (2022) employed LGCM to analyze the dynamic trajectories of foreign language reading strategy use. Their findings revealed a significant overall increase in strategy use over time, with notable individual variations in initial levels but no clear prediction of change rates. This aligns with CDST's focus on variability and the evolving nature of learning strategies within complex systems.

These findings collectively demonstrate the value of CDST in exploring the interplay between self-efficacy and strategy use. By addressing this gap, we aim to contribute to a more comprehensive understanding of the developmental processes underlying foreign language reading in EFL learners.

### 2.5 The present study

The existing research indicates that current studies on emotional dynamics in foreign language learning primarily focus on learners' overall self-efficacy, with a lack of dynamic examination of self-efficacy in specific foreign language skills. Furthermore, dynamic investigations into the development and changes in foreign language learning strategies remain scarce. There is an even greater deficiency of longitudinal dynamic research that simultaneously explores foreign language learning emotions and strategies, as well as the mutual predictive relationship between them.

As reviewed above, though foreign language reading self-efficacy and reading strategy use appears to be dynamic, most studies are cross-sectional and little longitudinal research can be found. The prior studies mainly focus on the comparison between the pre-test and post-test scores during one semester in investigating the changes in readers' self-efficacy and strategy use. There is little knowledge on how foreign language learners' reading self-efficacy and reading strategy use develop when observed over a longer period of time. The dynamic interactions between the two variables remain underexplored, too. Additionally, most studies examining foreign language reading self-efficacy are solely based on self-reported survey data. Qualitative data might have helped us better understand how foreign language reading self-efficacy changed in individual students and what factors caused the changes. Hence, the present study conducted five longitudinal surveys during one academic year to investigate Chinese English learners' foreign language reading self-efficacy and their use of foreign language reading strategies by employing a mixed-methods research design including parallel latent growth models, cross-lagged analysis, and semi-structured interviews. This study aims to explore the developmental trajectories and reciprocal predictive relationships between foreign language reading self-efficacy and reading strategy use. Specifically, it seeks to answer the following research questions:

(1) How do the students' foreign language reading self-efficacy and reading strategy use change throughout the year?
(2) What dynamic associations are observed between foreign language reading self-efficacy and reading strategy use?
(3) How does the students' foreign language reading self-efficacy predict their use of reading strategies, and vice versa?

## 3 Methodology

### 3.1 Participants and procedures

This study employed a convenience sampling approach to select participants, since this method is practical and cost-effective (Etikan et al., 2015). Altogether 293 non-English major freshmen were chosen, all of whom were registered for an English reading course taught by the same teacher at a university in Wuhan, China. This was to exclude other external factors and to ensure consistency of the findings, since all the participants were at the same proficiency levels, faced similar challenges and received the same EFL education. Upon enrollment to the university, all the freshmen took standardized foreign language placement tests, and based on their results (low, medium, and high), they were assigned to English reading courses of varying difficulty. The participants in this study were at an intermediate level of English proficiency, with an average of 9 years of English learning (starting from the third grade of primary school). The teacher taught 90 min per week during the 24-week English reading course. There was no attrition among the participants during the study period, and the final analysis included 197 male and 96 female students, with an average age of 19.10 years (SD = 0.64).

Informed consent was obtained from all participants. They were thoroughly briefed on the study's objectives, their role in the research, and any potential risks or benefits involved. They were also assured that their involvement was completely voluntary and that they could withdraw from the study at any time without facing any negative repercussions. Strict privacy and confidentiality protocols were maintained, ensuring all identifiable data was securely stored and exclusively accessible by the first author. Pseudonyms were employed throughout research documentation and in the presentation of findings.

These students participated in five waves of longitudinal measurements over the course of one academic year. According to Curran et al. (2010), a minimum of three repeated measures seemed adequate for growth models like LGCM. The English reading course lasted for a total of 24 weeks. To improve the accuracy of model fitting, the five measurements were conducted at equal intervals (at the 1^st^, 6^th^, 11^th^, 16^th^, and 21^st^ weeks). The five equal intervals were selected to capture developmental trends over a semester, aligning with typical progress markers in the English reading course. Firstly, the five equally spaced measurement intervals were chosen to mirror the instructional and developmental rhythms of the academic year. Positioning the five measurement points at equal intervals aligned with the gradual introduction and reinforcement of reading instruction and strategy training over the course. Secondly, this design enhanced the analytical precision of the growth models. By spacing the measurements evenly, the study ensured a consistent temporal framework to trace the changes in foreign language reading self-efficacy and reading strategy use. The participants were invited to complete online questionnaires via Wenjuanxing, a popular online survey platform in China. In order to mitigate response fatigue due to repeated questionnaire administration, the questionnaires were designed to be concise while maintaining data quality. Only essential items were included, avoiding unnecessary repetition and lengthy or complex questions. This approach ensured a manageable completion time and reduced participant burden. To sustain motivation, participants were assured of the confidentiality of their responses, reminded of the importance of their contributions, and offered research findings and feedback as a form of acknowledgment. After removing those with incomplete responses, 1,400 questionnaires remained, yielding a 95.56% valid response rate. To shed more lights on and provide deeper insights into the findings of the quantitative data, we took advantage of a qualitative phase along with the quantitative one and invited four students (two males and two females) to participate in the subsequent interviews after the administration of each questionnaire. All participants selected for the interviews were assigned pseudonyms (e.g., Student A, Student B, Student C, and Student D). Their real names and any personally identifiable details were not recorded in the transcripts. Additionally, code numbers were used in stored data files, ensuring that there was no direct link between the participants' identity and their responses.

### 3.2 Instruments

#### 3.2.1 Foreign language reading self-efficacy questionnaire

A questionnaire was designed to assess students' foreign language reading self-efficacy. The 11 items were adapted from the reading self-efficacy questionnaires developed by Kassem (2013) and Oh (2016). The scale used in this study had five factors constituting the self-efficacy construct: progress (2 items), observational comparison (2 items), strategic awareness (2 items), physiological states (2 items), and confidence in completing reading tasks (3 items). All items were scored on a 5-point Likert scale (1 = strongly disagree, 2 = disagree, 3 = undecided, 4 = agree, 5 = strongly agree), with higher scores indicating greater reading self-efficacy. The questionnaire was translated into Chinese and double-checked by the researchers proficient in both Chinese and English and proved to be highly reliable in the present research (Cronbach alpha = 0.858, 0.881, 0.880, 0.884, and 0.866 at time point 1, 2, 3, 4, 5 respectively). The results of confirmatory factor analysis (CFA) revealed that the scale exhibited strong structural validity (χ^2^ = 98.419, CFI = 0.946, TLI = 0.955, RMSEA = 0.043). A pilot test was conducted among 30 EFL students who were asked to comment on the contents of the questionnaire. As a result of the feedback received, minor adjustments were made to the wording. To enhance its external validity, the questionnaire was reviewed by two professors who taught English reading courses at the university.

#### 3.2.2 Reading strategy questionnaire

The 16-item reading strategy questionnaire was adapted from Shang (2010) to evaluate the frequency of students' self-reported strategy use. The adapted and translated scale were validated through expert review, pilot tests with a small sample size (30 students), and confirmatory factor analysis (χ^2^ = 288.527, CFI = 0.981, TLI = 0.979, RMSEA = 0.011). The questionnaire contained three major categories of reading strategies: cognitive (7 items), metacognitive (6 items), and compensation strategies (3 items). Students were asked to rate certain statements on a 5-point scale from 1 (never or almost never true of me) to 5 (always or almost always true of me). The higher the score, the more frequent the use of reading strategies. The reliability coefficient values for the scales at five time points are 0.855, 0.884, 0.879, 0.877, and 0.868 respectively.

#### 3.2.3 Semi-structured interviews

The data in the qualitative phase was collected via semi-structured interviews. Four students (see Table 1) were interviewed to ensure an in-depth and consistent analysis of individual developmental trajectories. Repeated interviews with the same participants at five points in time enabled us to trace changes in their foreign language reading self-efficacy throughout the year as well as the factors responsible for these changes. Despite the small size of interviewees, the present study could focus more on depth other than breadth, as detailed analysis of individuals could represent comprehensive EFL learning settings. Each interview, conducted in Chinese (students' L1), was about 20 min and transcribed into English.

**Table 1:** Information of interviewees.

**Student**	**Age**	**Gender**
A	19	Male
B	19	Male
C	19	Female
D	18	Female

### 3.3 Data collection and analysis

During the one-year English reading classes, the reading self-efficacy questionnaires, the reading strategy questionnaires, and student interviews were conducted at the 1^st^, 6^th^, 11^th^, 16^th^, and 21^st^ weeks respectively. The interval between the five measurements was 5 weeks, and the procedures for the follow-up measurements were consistent. The duration of each measurement was approximately 20 min on average. The questionnaires were in Chinese, translated and verified by the researchers from the original English. The quantitative data were analyzed using SPSS 26.0 and Mplus 8.3. SPSS 26.0 was used to standardize the data, compute the means and standard deviations of the measured variables, analyze the reliability of questionnaires, and examine correlations on the measured variables. Mplus 8.3 was applied to construct parallel latent growth models and run cross-lagged regression analyses.

In the qualitative stage, this study employed the three-tier coding approach of open coding-axial coding-selective coding proposed by Corbin and Strauss (1990) to systematically analyze the interview data. The analysis was conducted step by step using NVivo 11 software. The first stage of data analysis was open coding, which focused on labeling, conceptualizing, and categorizing the data. For example, the label “Positive evaluation of students' reading ability by teachers” was assigned to the statement, “My English teacher provided encouraging feedback on my assignment in the reading class, which gave me much confidence about my reading abilities”. A total of eight categories were identified at this stage. Axial coding delved deeper into the logical connections among categories and reorganized the initial categories to develop overarching themes. In this study, the eight categories were ultimately grouped into two main categories based on their logical and intrinsic relationships. Selective coding, the third stage of data collection and analysis, was used to identify the core categories connected to the main categories. This stage also involved developing a storyline to construct a theoretical model explaining the factors influencing changes in the interviewees' foreign language reading self-efficacy over the course of the year. The coding framework is shown in Table 2. To ensure intercoder reliability, the two authors independently analyzed and coded the same interview content, and the consistency of the resulting data or coding analysis was assessed through comparison. In addition, we sent the extracted themes to the interviewees in a timely manner to ensure that our interpretation of the data was consistent with what they really meant.

**Table 2:** The coding framework of the interview data.

**Open coding**	**Axial coding**	**Selective coding**
Students' sense of achievement after completing specific reading tasks	Category 1: accumulation of personal success	Core category 1: mastery experience
Increased confidence following improved reading comprehension accuracy		
Positive reinforcement from high scores in reading sections of exams		
Teachers assigning reading materials at an appropriate level to build students' confidence		
Influence of others' successful reading experiences (e.g., peers or senior students)	Category 2: influence of interactions with others	Core category 2: social persuasion
Improved reading ability through learning from classmates		
Positive evaluation of students' reading ability by teachers		
Supportive feedback from classmates boosting reading confidence		

## 4 Results

### 4.1 Descriptive statistics

Skewness and kurtosis values indicated that the data formed a normal distribution (Gravetter et al., 2020). Table 3 presents the overall situation of participants' foreign language reading self-efficacy and reading strategy use across five measurements and Pearson correlation analysis results. As shown in Table 3, the participants generally experienced medium levels of foreign language reading self-efficacy and reading strategy use (*M* ≥ 2.82). The participants' foreign language reading self-efficacy and reading strategy use were significantly positively correlated at all the five time points (*r* = 0.61 ~ 0.90, *p* < 0.001), which means that the higher the participants' foreign language reading self-efficacy, the higher the frequency of their foreign language reading strategy use, and vice versa. Reading self-efficacy measured at the five time points was significantly positively correlated with one another, with coefficients ranging from 0.61 to 0.87 (*p* < 0.001), and so was the students' reading strategy use at the five time points (*r* = 0.64 ~ 0.90, *p* < 0.001).

**Table 3:** Descriptive statistics and correlations of measures.

**Variable**	**Time**	**M**	**SD**	Correlation coefficients^*^									
Self-efficacy	T1	2.82	0.26	1.00									
	T2	3.09	0.19	0.74	1.00								
	T3	3.27	0.17	0.65	0.87	1.00							
	T4	3.18	0.22	0.68	0.80	0.81	1.00						
	T5	3.36	0.15	0.61	0.68	0.72	0.84	1.00					
Strategy	T1	2.94	0.12	0.79	0.81	0.74	0.72	0.63	1.00				
	T2	3.06	0.23	0.77	0.90	0.84	0.79	0.70	0.89	1.00			
	T3	3.19	0.10	0.72	0.86	0.87	0.85	0.78	0.79	0.89	1.00		
	T4	3.25	0.18	0.66	0.79	0.79	0.88	0.84	0.72	0.79	0.90	1.00	
	T5	3.44	0.13	0.61	0.72	0.71	0.79	0.86	0.64	0.72	0.81	0.90	1.00

* *p* < 0.001; Self-efficacy, foreign language reading self-efficacy; Strategy, foreign language reading strategy use; T1/T2/T3/T4/T5, time point 1/2/3/4/5.

Overall, foreign language reading self-efficacy demonstrated a trend of initial rise, followed by a decline, and then another rise: it increased from T1 to T3, decreased from T3 to T4, and increased from T4 to T5, with a significant increase from T1 to T5 (T1 = 2.82, T2 = 3.09, T3 = 3.27, T4 = 3.18, T5 = 3.36). The use of foreign language reading strategy showed an upward trend, with a significant increase from T1 to T5 (T1 = 2.94, T2 = 3.06, T = 3.19, T4 = 3.25, T5 = 3.44).

### 4.2 Developmental trajectories of foreign language reading self-efficacy and reading strategy use

**Figure 1:**
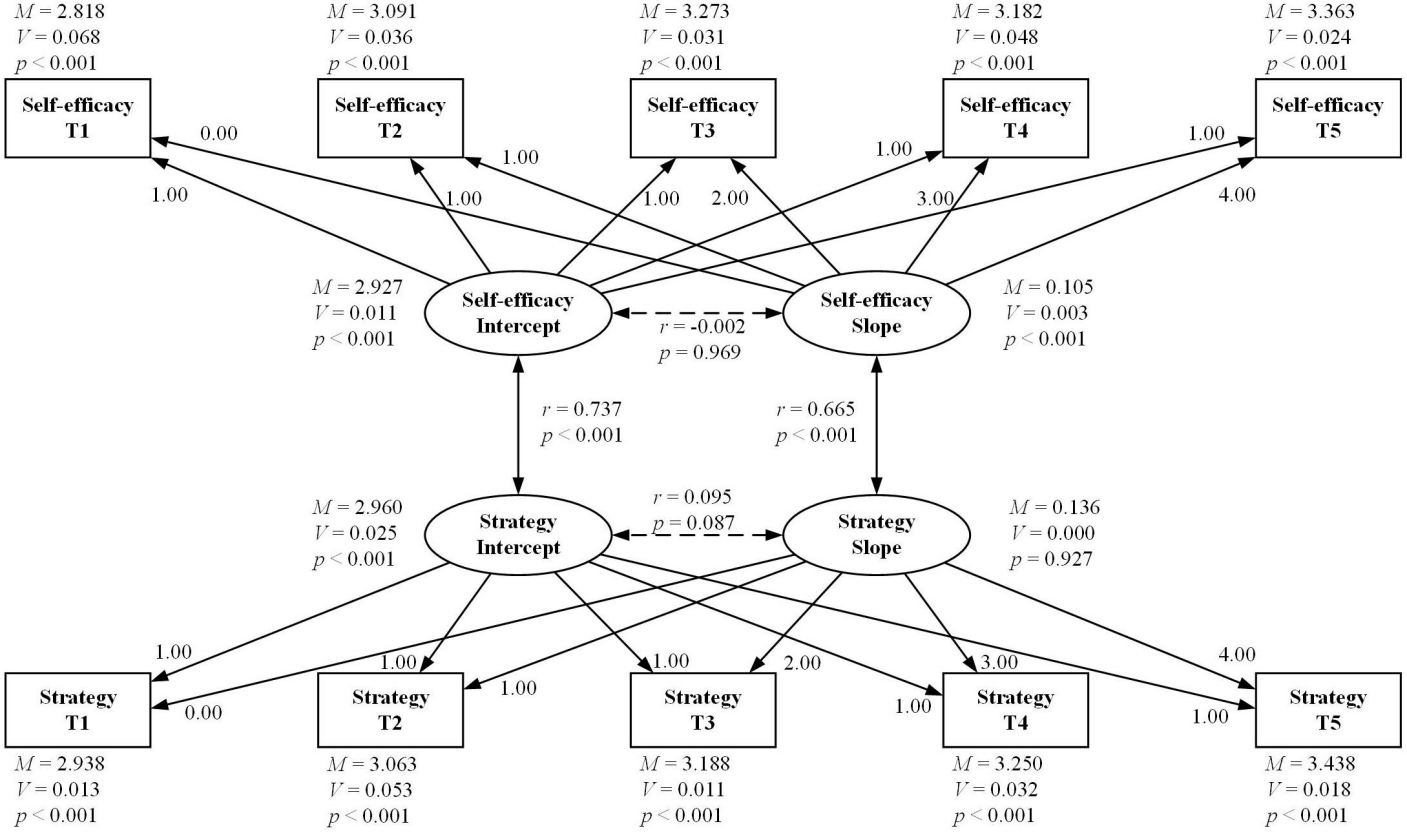
Developmental trajectories of foreign language reading self-efficacy and reading strategy use. M, mean; V, variance.

Figure 1 describes the participants' developmental trajectories of foreign language reading self-efficacy and reading strategy use measured at the five time points (The goodness-of-fit statistics for this model were χ^2^ = 20.02, *df* = 24, CFI = 1.00, TLI = 1.00, SRMR = 0.009). As shown in Figure 1, the participants' mean of initial reading self-efficacy was 2.927 with the variation being significant (*V* = 0.011, *p* < 0.001), implying that reading self-efficacy scores varied individually at a medium level. The slope of reading self-efficacy was 0.105 (*p* < 0.001), indicating that the participants' foreign language reading self-efficacy showed a significantly upward trend across the five time points. The variation in the slope of reading self-efficacy was significant (*V* = 0.003, *p* < 0.001), which meant that there were significant differences in the changing rates of the participants' reading self-efficacy over the year. The heterogeneity at both the initial stage as well as within the academic year was supported by the qualitative phase of the study. In the interviews, student A reported that he felt highly self-efficacious in T1 while student B experienced a low level of self-efficacy in the same phase. Likewise, in T4, student A and student B went through different trajectories with A experiencing low self-efficacy and B high self-efficacy. The heterogeneity in the slope can also be supported by student C and student D's self-efficacy trajectory in T2 with C and D going through high self-efficacy and low self-efficacy respectively. Additionally, the correlation (-0.002) between the intercept and the slope was weak, indicating that individuals' initial reading self-efficacy did not affect their rates of change. This disparity between initial reading self-efficacy and its growth patterns over the five time points was also evident in the interviews. Both student B and student C reported low reading self-efficacy in T1. However, while student C's self-efficacy significantly increased in T2, student B's did not. Student C attributed this boost to her teacher's positive feedback on her reading assignment. Bandura (1997) hypothesized that verbal persuasion—others' perception of one's capability to overcome a task—was a possible source of changes in individuals' self-efficacy. In T2, Student C became highly self-efficacious after receiving positive remarks from her teacher on an assignment. This supported the assumption that significant others could enhance one's reading self-efficacy (Elahi Shirvan et al., 2018).

Meanwhile, Figure 1 shows that the participants' initial mean of reading strategy use was 2.960 with the variation being significant (*V* = 0.025, *p* < 0.001), suggesting that significant differences existed in English reading strategy use among the participants. The slope of reading strategy use was 0.136 (*p* < 0.001), signifying a significant rise in the participants' reading strategy use across the five time points. Nevertheless, the variation in the slope of reading strategy use was not significant (*V* = 0.000, *p* = 0.927), indicating no significant heterogeneity in the developmental patterns of the participants' reading strategy use over the year. The model also demonstrated that the intercept was not correlated with the slope (*r* = 0.095, *p* = 0.087), implying that the students' initial use of reading strategies did not influence the rate of improvement.

Moreover, there was a positive correlation (*r* = 0.737, *p* < 0.001) between the intercepts of foreign language reading self-efficacy and reading strategy use, meaning that the participants with higher initial reading self-efficacy tended to use reading strategies more intensively. The result of the slopes also suggested that the growth pattern of reading self-efficacy was positively related to that of reading strategy use (*r* = 0.665, *p* < 0.001).

### 4.3 Cross-lagged regression analysis of foreign language reading self-efficacy and reading strategy use

**Figure 2:**
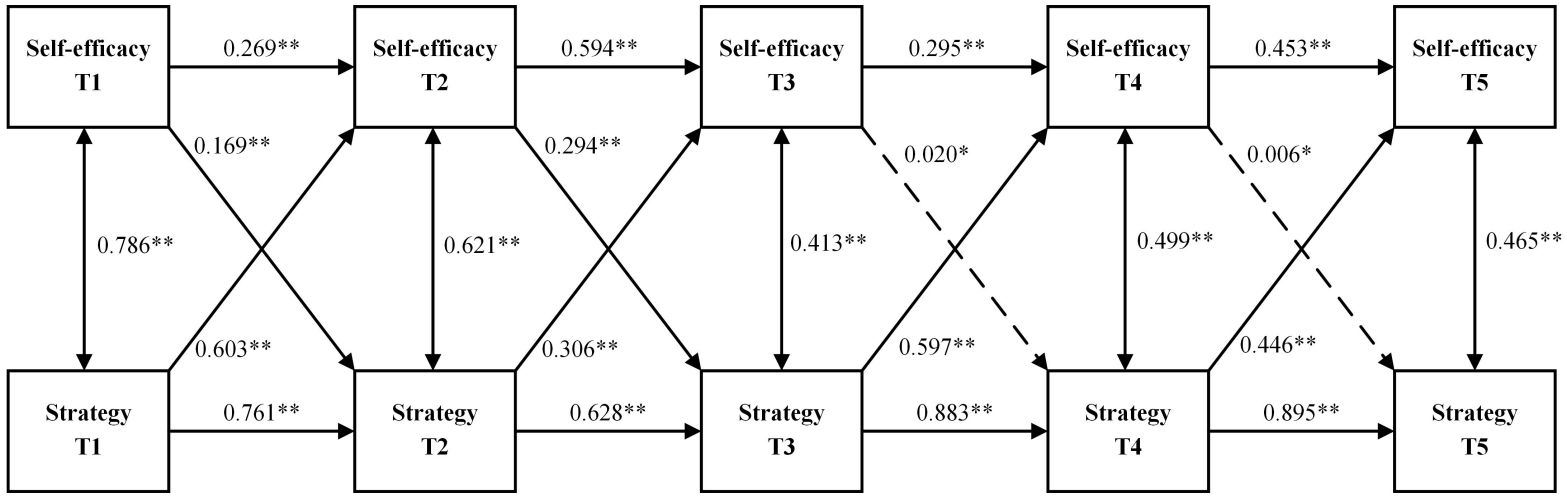
Cross-lagged regression analysis of reciprocal predictive relations between foreign language reading self-efficacy and reading strategy use. *p > 0.05, **p < 0.001.

This study employed Mplus 8.3 to develop a cross-lagged regression model between foreign language reading self-efficacy and reading strategy use (Figure 2). In this model, both foreign language reading self-efficacy and the frequency of reading strategy use served as predictors for subsequent levels. Figure 2 displays the prediction of reading strategy use based on foreign language reading self-efficacy and the prediction of foreign language reading self-efficacy based on the intensity of reading strategy use.

The model showcases that reading self-efficacy significantly positively predicted its value measured at the next time point (β = 0.269 at T1, *p* < 0.001, 0.594 at T2, *p* < 0.001, 0.295 at T3, *p* < 0.001, and 0.453 at T4, *p* < 0.001). The same was true for reading strategy use (β = 0.761 at T1, *p* < 0.001, 0.628 at T2, *p* < 0.001, 0.883 at T3, *p* < 0.001, and 0.895 at T4, *p* < 0.001). Across five measurements, reading self-efficacy positively predicted the frequency of reading strategy use at the next time point, but the predictive power was only significant at the initial two phases (β = 0.169 at T1 and 0.294 at T2, *p* < 0.001) and declined to non-significance from T3 onwards (*p* > 0.05). Meanwhile, reading strategy use positively predicted reading self-efficacy at the next time point, with the predictive effect being significant at all phases (*p* < 0.001).

## 5 Discussion

The present longitudinal study investigated the changes in foreign language reading self-efficacy and reading strategy use at five different time points during one academic year. Additionally, it examined the predictive relationships between these two variables.

### 5.1 Overall level and developmental trajectories of foreign language reading self-efficacy and reading strategy use

This study indicated that the participants generally experienced a medium level of foreign language reading self-efficacy and reading strategy use. Moreover, the positive and significant correlation between reading self-efficacy and reading strategy use observed in this study supports previous findings. For instance, Shang (2010) and Li and Wang (2010) both argued that individuals with higher reading self-efficacy tended to employ more reading strategies, and vice versa. These results highlight the dynamic interplay between self-efficacy and strategy use, consistent with prior cross-sectional research.

The present study also revealed that the participants' foreign language reading self-efficacy increased from T1 to T3, then decreased from T3 to T4, and again increased from T4 to T5, which implied that reading self-efficacy was not a stable trait, but rather to be formulated and developed across time. This finding highlights the limitations of cross-sectional studies on reading self-efficacy and underscores the need for more longitudinal research on the topic.

The heterogeneity in both initial levels and developmental trajectories of reading self-efficacy was supported by the qualitative phase of the study. During the interviews, Student A expressed a strong sense of self-efficacy in T1, while Student B reported a low level of confidence in their reading abilities at the same stage. Similarly, in T4, their trajectories diverged, with Student A experiencing a decline in self-efficacy and Student B showing an increase. The variation in self-efficacy trajectories was further supported by Student C and Student D's experiences in T2, where Student C reported a sharp increase in self-efficacy, while Student D's remained low.

The interview data also provided explanations for these fluctuations. For instance, Student C attributed her self-efficacy boost in T2 to her teacher's positive feedback on a reading assignment:

I was really happy when my teacher told me that my reading summary was well-structured and insightful. Before that, I always doubted my reading ability, but this gave me confidence. I started believing that I could actually improve.

This finding aligns with Bandura's (1997) hypothesis that verbal persuasion—feedback from significant others—can enhance self-efficacy (Elahi Shirvan et al., 2018). The qualitative data further explained the decline in reading self-efficacy from T3 to T4. The interviewees attributed this drop to the summer vacation, during which they had little exposure to English reading:

During the summer break, I didn't read much English. When the new semester started, I felt that my reading ability had declined. I wasn't as confident as before (Student D).

This suggests that continuous engagement with English reading materials, even outside the classroom, is crucial for maintaining self-efficacy. A possible pedagogical implication is that teachers should provide students with opportunities for structured reading practice during semester breaks, such as online reading tasks or recommended reading lists.

Additionally, one interviewee linked his decreased self-efficacy in T4 to his poor performance in an English exam at T3:

I thought I was doing well in reading, but my exam score was lower than expected. It made me doubt my ability to understand English texts (Student A).

As Bandura points out, mastery experience is one of the sources of possible change in individuals' self-efficacy (Elahi Shirvan et al., 2018). As a strong source of self-efficacy belief, mastery experience indicates the positive effect of prior successful performances. In other words, accomplishment in performances such as examinations increases individuals' self-efficacy while failure in them reduces their senses of self-efficacy.

This study also found significant changes in reading self-efficacy across the five time points and significant differences in its growth patterns among the participants (see Figure 1). Figure 1 shows significant differences among the participants in both initial levels and rates of change in reading self-efficacy, highlighting the heterogeneity in foreign language learners' reading self-efficacy (Oh, 2016). Furthermore, the present study revealed that the initial levels of reading self-efficacy did not influence its growth rate. In other words, a lower initial level of reading self-efficacy did not necessarily lead to a slower growth rate, consistent with the findings in Elahi Shirvan et al. (2018) and Kyo (2022) regarding English learners in Iran and South Korea. This was also evident in the interview data:

At first, I wasn't confident in reading at all. But after practicing with different strategies, I started feeling more capable, even though I wasn't sure at the beginning (Student C).

The results imply that reading self-efficacy is malleable and can be improved through means of interventions (Yang and Gan, 2024). Accordingly, foreign language teachers should actively assist learners in building confidence during the teaching process, helping them recognize that although their current self-efficacy may be limited, improving it is both feasible and highly effective. The heterogeneity observed in the developmental trajectories of the participants can be justified under the influence of some other variables as learners' individual differences or related to the classroom ecology. The qualitative data from interviews revealed that mastery experience and verbal persuasion were the important factors affecting the students' reading self-efficacy. As suggested by previous researchers (e.g., Klassen, 2004), cultural dimensions powerfully influence the formation of efficacy beliefs. For Asian students, self-efficacy is more profoundly influenced by the other-oriented sources such as verbal persuasion (Klassen, 2004). This is largely due to a greater emphasis on social comparison and social hierarchy, which leads to their self-efficacy being shaped to a much greater extent by others compared to students from individualistic cultural contexts. Specifically, in Chinese education settings, the teacher-student power distance and collectivism norms significantly shape the interpretation and impact of verbal persuasion (He and Zhang, 2024). Teachers, often regarded as the ultimate authority in the classroom, are seen as the primary source of knowledge and evaluation. This cultural sanctioning of teacher authority means that verbal persuasion, such as praise or encouragement, is perceived not merely as personal opinion but as an authoritative validation of students' abilities. Consequently, positive feedback from teachers can have an outsized influence on boosting self-efficacy, as students equate such feedback with objective confirmation of their competence. Additionally, students from cultural backgrounds with rather collectivist orientations have reported verbal persuasion as the most important source of self-efficacy (Ahn et al., 2017). In collectivist cultures, others are assigned significantly more importance, their influence is greater, and they are considered central in shaping an individual's behavior. The collectivist emphasis on group harmony and shared goals may amplify the reach of verbal persuasion beyond the individual. For instance, when a teacher praises one student's effort or success, it is often perceived as a reflection of the entire group's capability, thereby elevating the self-efficacy of the classroom community as a whole. This dynamic contrasts with individualistic cultures, where verbal persuasion may be interpreted as relevant only to the individual receiving the feedback, limiting its broader influence.

In terms of foreign language reading strategies, this study found that the use of foreign language reading strategies among the participants demonstrated a significant upward trend across the five time points. This improvement may be attributed to the fact that, in foreign language teaching, learners' use of reading strategies tends to increase as they acquire more knowledge and receive more instruction from teachers (Lien, 2016). While the initial level of reading strategy use varied significantly among individuals, no significant individual differences were observed in the rates of change. Besides, the initial level of reading strategy use did not influence the rates of improvement. These findings support the necessity and effectiveness of reading strategies instruction. Foreign language teachers should not only hold a strong belief in the value of teaching reading strategies but also take active roles in helping learners develop their own belief in enhancing reading strategies.

Moreover, the parallel latent growth model indicated that the participants with higher initial reading self-efficacy tended to use more reading strategies. The increase in reading self-efficacy was significantly associated with the increase in reading strategy use and vice versa. These findings suggest beneficial impact of reading strategy use on improving foreign language learners' reading self-efficacy, as observed in several cross-sectional surveys (Fathi and Soleimani, 2020; Liao and Wang, 2018). Since these results have been seldom reported in the current literature, more research is needed.

### 5.2 The reciprocal predictive relationships between foreign language reading self-efficacy and reading strategy use

Cross-lagged regression analyses indicated that reading self-efficacy positively predicted the frequency of reading strategy use at the next time point, consistent with the results in Shang (2010) and Li and Wang (2010). But the present study found that this predictive effect was only significant at T1 and T2 and then weakened to non-significance from T3 onwards. According to Li and Wang (2010), foreign language readers with high self-efficacy are likely to engage in making reading plans, apply cognitive strategies, assess their reading performance, and manage negative emotions when facing reading difficulties. The inability of reading self-efficacy to predict reading strategy use for a longer time may stem from students' growing foreign language learning experience. As their experience increases, their attitudes, emotions, and motivations toward foreign language reading may become more diverse. These factors can variably influence learners' use of reading strategies, highlighting the need for further research.

Moreover, at all the five time points, reading strategy use significantly predicted reading self-efficacy in the subsequent phase. This suggests a bidirectional relationship between foreign language reading self-efficacy and strategy use, with the impact of reading strategy on self-efficacy being stronger than the reverse. Given the rarity of such findings in the current literature, additional research is needed to explore this area more thoroughly. It should be also noted that, some external factors such as variations in motivation and extracurricular activities were not directly controlled but may represent important variables for future research to examine their influence on self-efficacy and strategy use. For instance, initial level of EFL students' motivation could be a predictor of the rate of their self-efficacy change (Elahi Shirvan et al., 2018). Extracurricular reading activities including traditional book reading and online chatting might affect students' reading comprehension (Pfost et al., 2013).

## 6 Conclusion

Drawing on both quantitative and qualitative data obtained from 293 EFL undergraduates in five longitudinal surveys, this study explored the developmental trajectories and reciprocal predictive relationships between Chinese university students' foreign language reading self-efficacy and reading strategy use. The findings revealed that (1) the participants' foreign language reading self-efficacy and reading strategy use were both at moderate levels. Foreign language reading self-efficacy showed a trend of rising initially, then declining, and rising again across the five measurements, with significant individual differences in both initial levels and rates of change. The initial levels did not affect the rates of increase. In terms of foreign language reading strategy use, it showed an upward trend. Although there were significant individual differences in the initial levels, no significant individual differences were observed in the rates of increase, and the initial levels did not influence the rates of growth. (2) The initial levels and growth patterns of reading self-efficacy were positively correlated with those of reading strategy use. (3) A bidirectional predictive relationship between foreign language reading self-efficacy and strategy use was identified, with the influence of reading strategy use on self-efficacy being more substantial than that of self-efficacy on strategy use. Reading self-efficacy positively predicted the frequency of reading strategy use at the next time point, but this predictive effect was only significant in the initial stages. Reading strategy use consistently and significantly predicted reading self-efficacy in the subsequent phases.

The above-mentioned research findings can provide valuable pedagogical implications. First of all, this study offers important insights into the dynamics of foreign language reading self-efficacy and reading strategy use among adult learners of English. In the context of adult EFL reading instruction, which is a key focus within applied linguistics, the study demonstrates that learners often experience fluctuations in their reading self-efficacy and use of reading strategies. These fluctuations should be taken into account when considering pedagogical aspects such as reading instruction curriculum design and evaluation. Adult EFL learners' reading performance should not be solely assessed based on early assignments or activities in the course. Instead, continuous monitoring and evaluation are essential to capture their cognitive and emotional development in reading skills. Raising awareness among EFL reading educators about the dynamic nature of learners' reading self-efficacy and reading strategy use can help them select more effective assessment methods, ultimately guiding students' progress in developing their reading skills. Secondly, since reading self-efficacy is malleable and can be enhanced by means of interventions, teachers are recommended to implement self-efficacy-enhancing instructions in their classrooms to help students form more positive assessments of their reading abilities. This is particularly important in Asian contexts, where students often tend to be conservative and underestimate their capabilities (Scholz et al., 2002). For example, teachers can make reading strategies explicit to students, allowing them to gain greater control over their reading processes (Graham et al., 2020; Li et al., 2022). Teachers are also encouraged to create a more supportive reading atmosphere in the classroom where students can receive positive feedbacks from peers and teachers so that students' confidence in completing their reading tasks will be improved (McElvain, 2010). Thirdly, the strong predictive effect of reading strategy use on reading self-efficacy suggests the necessity and effectiveness of incorporating reading strategy use instructions. Encouraging the teaching of reading strategies in English classes is crucial, as it can boost foreign language learners' reading self-efficacy.

Although this research has provided valuable insights into foreign language reading self-efficacy and reading strategy use, it is essential to acknowledge certain limitations. Firstly, the subjects of this study were 293 undergraduate EFL students from a single Chinese university. Thus, the findings may not be generalizable to a wider pool of participants from diverse contexts. Secondly, the approaches employed in this study may affect the generalizability of the findings due to their limitations. For example, the convenience sampling approach may have restrained representativeness and sampling bias, hence the future studies will conduct random sampling in various teaching contexts to verify the present research. While self-reported questionnaire can reflect students' individual learning conditions, it may be subjective and invites potential biases. Future research should consider complementing self-reports with performance-based assessments or observational data to obtain objective measures of reading skills. Thirdly, a deeper and more comprehensive understanding on the developmental trajectories and reciprocal predictive relationship between foreign language reading self-efficacy and reading strategy use can be achieved by tracking changes in important variables, such as aspects of reading self-efficacy—including social comparison influences and controllability over progress, observational comparison, physiological states—and aspects of reading strategy use, which encompass cognitive, metacognitive, and compensation strategies. Lastly, future research can examine certain contextual factors that may influence foreign language learners' reading self-efficacy and reading strategy use, such as the design of reading courses, classroom environment, and teacher's support.

## Data Availability

The raw data supporting the conclusions of this article will be made available by the authors, without undue reservation.
