# Supplementary material for: Developmental trajectories of and reciprocal relationships between Chinese university students' foreign language reading self-efficacy and reading strategy use
Source: Front Psychol. 2025 Apr 11;16:1512098. doi: 10.3389/fpsyg.2025.1512098 (PMC12023475; doi:10.3389/fpsyg.2025.1512098)
Supplement: Supplementary file 1 [file Supplementary_file_1.docx]

**Appendix: Reading Self-Efficacy and Reading Strategy Use Questionnaires**

Student Number_________________________________

**Part One - Reading Self-Efficacy Questionnaire**

Please read the following questions carefully and make an accurate evaluation of your reading abilities. These questions are designed to measure your judgment of your capabilities, so there are no right or wrong answers. Please choose the number accurately representing your capabilities.

1. I read better now than I could before.

- 1. Strongly Disagree
- 2. Disagree
- 3. Undecided
- 4. Agree
- 5. Strongly Agree

1. I believe that my reading comprehension improves with time.

- 1. Strongly Disagree
- 2. Disagree
- 3. Undecided
- 4. Agree
- 5. Strongly Agree

1. When I compare myself to other students in my class, I am a good reader.

- 1. Strongly Disagree
- 2. Disagree
- 3. Undecided
- 4. Agree
- 5. Strongly Agree

1. I am more confident in my reading than other students.

- 1. Strongly Disagree
- 2. Disagree
- 3. Undecided
- 4. Agree
- 5. Strongly Agree

1. Reading is a pleasant activity for me.

- 1. Strongly Disagree
- 2. Disagree
- 3. Undecided
- 4. Agree
- 5. Strongly Agree

1. I feel calm when I read and do reading assignments.

- 1. Strongly Disagree
- 2. Disagree
- 3. Undecided
- 4. Agree
- 5. Strongly Agree

1. I know what strategies to use when I read.

- 1. Strongly Disagree
- 2. Disagree
- 3. Undecided
- 4. Agree
- 5. Strongly Agree

1. I can make a plan about reading the text before I begin to read.

- 1. Strongly Disagree
- 2. Disagree
- 3. Undecided
- 4. Agree
- 5. Strongly Agree

1. I can read and understand the textbook passages from English classes.

- 1. Strongly Disagree
- 2. Disagree
- 3. Undecided
- 4. Agree
- 5. Strongly Agree

1. I can figure out the main ideas of English materials that I search on the Internet.

- 1. Strongly Disagree
- 2. Disagree
- 3. Undecided
- 4. Agree
- 5. Strongly Agree

1. I can read and figure out the main ideas of articles from English newspapers.

- 1. Strongly Disagree
- 2. Disagree
- 3. Undecided
- 4. Agree
- 5. Strongly Agree

**Part Two - Reading Strategy Questionnaire**

The following statements are about the strategies you use in reading English texts. Please indicate the frequency of reading strategies you use by choosing the following appropriate numbers.

1. I try to remember key words to understand the main idea of the text.

- 1. Never or almost never true of me
- 2. Usually not true of me
- 3. Somewhat true of me
- 4. Usually true of me
- 5. Always or almost always true of me

1. I underline key words to remind me of important concepts of the text.

- 1. Never or almost never true of me
- 2. Usually not true of me
- 3. Somewhat true of me
- 4. Usually true of me
- 5. Always or almost always true of me

1. When I read the text, I take notes by writing down the key words.

- 1. Never or almost never true of me
- 2. Usually not true of me
- 3. Somewhat true of me
- 4. Usually true of me
- 5. Always or almost always true of me

1. I go back to read the details of the passage for the answers of some questions.

- 1. Never or almost never true of me
- 2. Usually not true of me
- 3. Somewhat true of me
- 4. Usually true of me
- 5. Always or almost always true of me

1. I do not need to understand every detail in each text to get the main idea correctly.

- 1. Never or almost never true of me
- 2. Usually not true of me
- 3. Somewhat true of me
- 4. Usually true of me
- 5. Always or almost always true of me

1. Before I study new material thoroughly, I often skim it to see how it is organized.

- 1. Never or almost never true of me
- 2. Usually not true of me
- 3. Somewhat true of me
- 4. Usually true of me
- 5. Always or almost always true of me

1. I skim/scan in the appropriate part of the text for the key word or idea.

- 1. Never or almost never true of me
- 2. Usually not true of me
- 3. Somewhat true of me
- 4. Usually true of me
- 5. Always or almost always true of me

1. I read the topic or heading of the passage.

- 1. Never or almost never true of me
- 2. Usually not true of me
- 3. Somewhat true of me
- 4. Usually true of me
- 5. Always or almost always true of me

1. I read the first sentence of the passage.

- 1. Never or almost never true of me
- 2. Usually not true of me
- 3. Somewhat true of me
- 4. Usually true of me
- 5. Always or almost always true of me

1. I make sure that I keep up with the weekly readings and assignments for this course.

- 1. Never or almost never true of me
- 2. Usually not true of me
- 3. Somewhat true of me
- 4. Usually true of me
- 5. Always or almost always true of me

1. I notice my reading difficulties and try to use other methods to help me understand the text better.

- 1. Never or almost never true of me
- 2. Usually not true of me
- 3. Somewhat true of me
- 4. Usually true of me
- 5. Always or almost always true of me

1. I slow the pace of reading when confronting with more difficult texts.

- 1. Never or almost never true of me
- 2. Usually not true of me
- 3. Somewhat true of me
- 4. Usually true of me
- 5. Always or almost always true of me

1. I try to find out how to be a better reader of English.

- 1. Never or almost never true of me
- 2. Usually not true of me
- 3. Somewhat true of me
- 4. Usually true of me
- 5. Always or almost always true of me

1. To understand unfamiliar English words, I make guesses from suffixes and prefixes.

- 1. Never or almost never true of me
- 2. Usually not true of me
- 3. Somewhat true of me
- 4. Usually true of me
- 5. Always or almost always true of me

1. I predict what is going to happen next while reading.

- 1. Never or almost never true of me
- 2. Usually not true of me
- 3. Somewhat true of me
- 4. Usually true of me
- 5. Always or almost always true of me

1. I use my background knowledge to guess the overall meaning of the text.

- 1. Never or almost never true of me
- 2. Usually not true of me
- 3. Somewhat true of me
- 4. Usually true of me
- 5. Always or almost always true of me
